# Multi-Sensor Information Fusion Positioning of AUKF Maglev Trains Based on Self-Corrected Weighting

**DOI:** 10.3390/s25082628

**Published:** 2025-04-21

**Authors:** Qian Hu, Hong Tang, Kuangang Fan, Wenlong Cai

**Affiliations:** 1 School of Electrical Engineering and Automation, Jiangxi University of Science and Technology, Ganzhou 341000, China; 6720220742@mail.jxust.edu.cn (Q.H.); 6720230685@mail.jxust.edu.cn (W.C.); 2 Jiangxi Province Key Laboratory of Maglev Rail Transit Equipment, Jiangxi University of Science and Technology, Ganzhou 341000, China; 3 School of Intelligent Manufacturing and Materials Engineering, Gannan University of Science and Technology, Ganzhou 341000, China

**Keywords:** maglev trains, multi-sensor information fusion, high accuracy positioning, self-correcting weighting, AUKF

## Abstract

Achieving accurate positioning of maglev trains is one of the key technologies for the safe operation of maglev trains and train schedules. Aiming at magnetic levitation train positioning, there are problems such as being easily interfered with by external noise, the single positioning method, and traditional weighting affected by historical data, which lead to the deviation of positioning fusion results. Therefore, this paper adopts self-corrected weighting and Sage–Husa noise estimation algorithms to improve them and proposes a research method of multi-sensor information fusion and positioning of an AUKF magnetic levitation train based on self-correcting weighting. Multi-sensor information fusion technology is applied to the positioning of maglev trains, which does not rely on a single sensor. It combines the Sage–Husa algorithm with the unscented Kalman filter (UKF) to form the AUKF algorithm using the data collected by the cross-sensor lines, INS, Doppler radar, and GNSS, which adaptively updates the statistical feature estimation of the measurement noise and eliminates the single-function and low-integration shortcomings of the various modules to achieve the precise positioning of maglev trains. The experimental results point out that the self-correction-based AUKF filter trajectories are closer to the real values, and their ME and RMSE errors are smaller, indicating that the self-correction-weighted AUKF algorithm proposed in this paper has significant advantages in terms of stability, accuracy, and simplicity.

## 1. Introduction

Rail transit has the advantages of large capacity, high speed, low energy consumption, low pollution, high security, strong punctuality, etc., and it plays an important basic, supportive, and service role in the development of the national economy and people’s livelihood in society. Maglev technology has been applied in various fields such as wind turbines [1], space launches [2], centrifuges [3], lifts [4], cardiac pumps [5], and magnetic levitation bearings [6], accumulating a large amount of technological experience that can be used as a reference for magnetic levitation transport.

As a typical green, safe, efficient, and new type of public transport system [7], levitated permanent magnet maglev monorail transport is suitable for small and medium-capacity transport systems, especially for small cities, tourist attractions, airports, transport hubs, and other transport systems with relevant connections or feeder routes. The crucial requirement for ensuring the safe functioning of the entire system is the precise and prompt detection of a maglev train’s position and speed at a given moment. Thus, studying their location is important and necessary to lay the groundwork for the future development of maglev trains [8,9]. Nowadays, there are various problems, such as a single sensor, a simple positioning method, and being easily disturbed by the environment, which lead to poor robustness of information fusion, low positioning accuracy, and poor stability of the magnetic levitation train positioning [10]. A variety of sensors are used to collect data and information on maglev trains to locate them. Using multiple sources of information to complement each other can avoid the loss of information from a single sensor, environmental interference, and inability to accurately locate, ultimately improving the positioning accuracy of maglev trains [11].

Random errors or mistakes in sensors in multi-sensor systems can be caused by noise, electromagnetic interference between devices, and environmental conditions that are present in real-world applications [12]. This may lead to bias or even distortion in the measurement findings, which could result in the maglev train being positioned incorrectly [13]. By giving each sensor a weighting factor, weighted fusion [14,15] creates an ideal, unbiased fusion result that minimizes the mean-square error fusion result without needing prior knowledge of the system or observation noise [16,17]. Indeed, the distribution of weights has an important effect on weighted fusion performance. If the weights are not evenly distributed, the accuracy and reliability of the system may not be significantly improved [18]. Therefore, in weighted fusion, to ensure the accuracy of the estimation, the weights must be distributed properly.

UKF [19,20] is an algorithm for the state estimation of nonlinear systems, which is an extension of the traditional Kalman Filter (KF) and was proposed by Julier and Uhlmann in 1997 for solving estimation problems in nonlinear systems. The principle is to capture the mean and covariance information of a random variable using the Unscented Transform (UT) [21] and propagate this information through a nonlinear transformation. Throughout the UKF filtering process, the direct application of existing filtering algorithms suffers from filtering dispersion problems due to time-varying and unknown system measurement noise [22,23]. However, determining the covariance of the measurement noise is a major obstacle in practice. SAGE-HUSA filtering [24] is a combination of the SAGE and HUSA algorithms, which is suitable for dealing with the problem of state estimation of nonlinear systems and non-Gaussian noises, and the main idea is to utilize the average of the historical information to evaluate the current array of the system noise variance and the array of the measurement noise variance. The algorithm is solved using a Kalman filter [25] modeling algorithm to obtain the optimal estimation of the system state, which improves the accuracy and robustness of the state estimation. The UKF algorithm is more common in vehicle state estimation and train operation studies.

In [26], for the problem of degradation of positioning accuracy due to the interruption of satellite signals when the train passes through long tunnels, a train positioning method based on adaptive Unscented Kalman Filtering (UKF) is proposed, which takes into account the dynamic characteristics of the train and employs a sequence of innovations for the real-time estimation of the covariance of the measurement noise. In [27], a state observer design with an Adaptive Unscented Kalman Filter (AUKF) is proposed to address the effect of uncertain noise on vehicle state parameter estimation and the high cost of sensors; the effectiveness of the method is demonstrated through simulation and analysis. In [28], to address the challenge of the vehicle driving state observer being challenged by unknown measurement noise and transient perturbations caused by complex conditions and sensor failures, a modified Sage–Husa maximum posterior is used to correct for the measurement noise; the covariance moments are updated after the scatter is detected in order to minimize the impact of transient perturbations on the subsequent process.

Accurate positioning is critical for maglev train safety. However, single sensors often lack precision and reliability due to noise interference. By fusing data from multiple sensors, we achieve significantly higher positioning accuracy. However, for traditional weighting in the case of dynamically changing data, fixed weights may not accurately reflect the real situation of the current data, with certain deviations. Therefore, this paper proposes a self-correcting weighted AUKF research method. The main contributions of our work are as follows:

(1) A self-correcting weighting algorithm is proposed. The traditional weighting algorithm with fixed weights and susceptibility to historical data is improved by introducing an attenuation memory factor to reduce the influence of historical data.
(2) The Sage–Husa algorithm is introduced to adaptively update the statistical feature estimation of the measurement noise to effectively reduce the interference of noise, which is combined with the UKF algorithm to realize the precise positioning of the train.
(3) The improved UKF algorithm is applied to the positioning of maglev trains. The experimental results prove the effectiveness of the method and provide a reference for the positioning of maglev trains.

This is how the remainder of the paper is organized. Section 2 develops a mathematical model for the fusion of self-correcting weighting and adaptive UKF algorithms. Section 3 performs experiments and analyzes the results. Section 4 concludes the paper.

## 2. Model Building

### 2.1. Sensor Data Processing

The navigation system used in this experiment consists of a combination of relative and absolute positioning. The relative positioning system consists of three components, a GNSS receiver, a radar sensor, and an INS sensor, and the absolute positioning is provided by a cross-sensing line.

(1) GNSS positioning device

GNSS can obtain the latitude and longitude of the target The position information of the target can be obtained by iterative calculation of the latitude and longitude formula between two points, and the mileage of the maglev train is calculated using the latitude and longitude information, and the distance between neighboring latitudes and longitudes per unit time is calculated using Haversine’s formula:

(1) $$Hav(\theta )=\sin^{2}\left(\frac{\theta }{2}\right)=\frac{1-\cos\left(\theta \right)}{2}$$

(2) $$\begin{array}{ll} d & =2R\times \arcsin\left(\sqrt{Hav\left(\varphi_{B}-\varphi_{A}\right)+\cos\varphi_{A}\cos\varphi_{B}Hav\left(\theta_{B}-\theta_{A}\right)}\right)\\ & =2R\times \arcsin\left(\sqrt{\sin^{2}\left(\frac{\varphi_{B}-\varphi_{A}}{2}\right)+\cos\varphi_{A}\cos\varphi_{B}\sin^{2}\left(\frac{\theta_{B}-\theta_{A}}{2}\right)}\right) \end{array}$$

where R is the radius of the Earth, $\varphi_{A}$ and $\varphi_{B}$ are the latitudes of points A and B, $\theta_{A}$ and $\theta_{B}$ are the longitudes of points A and B, and d is the distance between points A and B.

(2) INS positioning device

INS measures the acceleration of a moving body; after a single integration of time, the velocity is obtained, and the velocity then undergoes a further integration of time to obtain the distance. INS achieves navigation and positioning based on:

(3) $$x\left(k\right)=x_{0}+\int_{0}^{k}v\left(k\right)dk$$

(4) $$v\left(k\right)=v\left(0\right)+\int_{0}^{k}a\left(k\right)dk$$

where $x_{0}$ is the initial position, v is the speed, a is the acceleration.

(3) Radar positioning device

The radar primarily picks up the velocity of the target, and a single integration of the velocity yields a displacement.

(5) $$v\left(k\right)=v\left(0\right)+\int_{0}^{k}a\left(k\right)dk$$

(4) Cross-sensing line

The cross-sensing line mainly collects the displacement of the target, which is calibrated in this experiment as an absolute positioning method and is the true value in the experiment.

### 2.2. Self-Correcting Weighted Fusion

Distributed fusion [29] algorithms feed individual local state estimates into the fusion center, which are weighted according to a certain fusion criterion to obtain a fusion estimate, whose weights determine the accuracy of the fusion. An enhanced weight calculation method is created by adding the decay memory factor. The conventional weight calculation method [30] is based on the cumulative deviation of all historical measurement signals, whose weights are influenced by historical data. The precise procedure is as follows:

(1) The center point of the sensor location ${\bar{Z}}_{k}$:

(6) $${\bar{Z}}_{k}=\frac{1}{M}\sum_{i=1}^{M}Z_{k}^{\left(i\right)}$$

where $Z_{k}^{\left(i\right)}$ is the measurement value of the ith sensor at time k, and M is the number of sensors in the multi-sensor system.

(2) Discrepancy of the measured value from the center point $\Delta Z_{k}^{\left(i\right)}$:
  (7) $$\Delta Z_{k}^{\left(i\right)}=Z_{k}^{\left(i\right)}-{\bar{Z}}_{k}$$

(3) The total of both the squared and sensor deviations:

(8) $$S_{k}^{\left(i1\right)}=\tau S_{k-1}^{\left(i1\right)}+\left(1-\tau \right)\Delta Z_{k}^{\left(i\right)}$$

(9) $$S_{k}^{\left(i2\right)}=\tau S_{k-1}^{\left(i2\right)}+\left(1-\tau \right){\left(\Delta Z_{k}^{\left(i\right)}\right)}^{2}$$

where $S_{k}^{\left(i1\right)}$ is the sum of the asymptotic memory deviations of the ith sensor at the kth moment, $S_{k}^{\left(i2\right)}$ is the sum of the squared asymptotic memory deviations of the ith sensor at the kth moment, and τ takes values in the range $\left[0,1\right]$.

(4) Mean value of the sensor’s deviance $\Delta {\bar{Z}}_{k}^{\left(i\right)}$:
  (10) $$\Delta {\bar{Z}}_{k}^{\left(i\right)}=S_{k}^{\left(i1\right)}/M$$

(5) Sensor bias standard deviation $\sigma_{k}^{\left(i\right)}$:
  (11) $$\sigma_{k}^{\left(i\right)}={\left[\frac{S_{k}^{\left(i2\right)}-2\Delta {\bar{Z}}_{k}^{\left(i\right)}S_{k}^{\left(i1\right)}+k{\left(\Delta {\bar{Z}}_{k}^{i}\right)}^{2}}{k-1}\right]}^{1/2}$$

(6) Calculate the weight of each sensor $\eta_{k}^{\left(i\right)}$:
  (12) $$\eta_{k}^{\left(i\right)}={\left[{\left(\sigma_{k}^{i}\right)}^{2}\sum_{i=1}^{M}\frac{1}{{\left(\sigma_{k}^{i}\right)}^{2}}\right]}^{-1}$$

To realize the fusion of multi-sensor information for permanent magnet maglev trains, a self-correcting weighted fusion estimation algorithm introduces an attenuation memory factor and a decrease in the weight of historical measurement data’s influence on the fusion estimate.

The formula for the weighted fusion algorithm is shown in Equation (13):

(13) $$Z_{kw}=\sum_{i=1}^{M}\left[\eta_{k}^{\left(i\right)}Z_{k}^{\left(i\right)}\right]$$

where $Z_{kw}$ is the weighted, measured value. The weights of each sensor’s measurements at each moment are calculated according to Equations (6)–(12), and the weighted measurements at the k moment are calculated using Equation (13), which are substituted into the adaptive UKF algorithm to obtain the final fusion estimation results.

### 2.3. Standard UKF

The process and measurement model, taking into account a generic nonlinear discrete-time dynamic system, can be explained as follows:

(14) $$\left\{\begin{array}{l} X_{k}=f\left(X_{k-1}\right)+\omega_{k-1}\\ Z_{k}=h\left(X_{k}\right)+\nu_{k} \end{array}\right.$$

where $X_{k}$ is the state vector of train position, $Z_{k}$ is the measurement vector of train position observations, $f\left(•\right)$ is the nonlinear state transfer function, $h\left(•\right)$ is the nonlinear measurement function, ω is the process noise, and ν is the observation noise.

The covariance of $\omega_{k-1}$ is $Q_{k-1}$ and the covariance of $\nu_{k}$ is $R_{k}$, which is satisfied:

(15) $$E\left(\omega_{k}\omega_{k}^{T}\right)=Q_{k},\;E\left(\nu_{k}\nu_{k}^{T}\right)=R_{k}$$

UKF is a nonlinear filtering method that estimates the state of a system that is not linear and has a high degree of accuracy and robustness when working with nonlinear systems. The following are the major steps:

Step 1: Initialisation

Step 2: Sigma Point Calculation

(16) $$\left\{\begin{array}{l} \chi_{k-1}^{\left(0\right)}={\hat{X}}_{k-1}\\ \chi_{k-1}^{\left(i\right)}={\hat{X}}_{k-1}+\sqrt{\left(n+\lambda \right)P_{k-1}},i=1,2,\cdots ,n\\ \chi_{k-1}^{i}={\hat{X}}_{k-1}-\sqrt{\left(n+\lambda \right)P_{k-1}},i=n+1,n+2,\cdots ,2n \end{array}\right.$$

where ${\hat{X}}_{k-1}$ is the predicted mean, $\chi_{k-1}^{\left(i\right)}$ is the sigma point mean, n is the dimensionality of the state, λ is the composite scale factor:

(17) $$\lambda =\alpha^{2}\left(n+\kappa \right)-n$$

where the parameter α is usually set to 0≤α≤1, and κ is usually set to 0 by default.

Step 3: State Prediction

(18) $$\chi_{k}^{\left(i\right)}=f\left(\chi_{k-1}^{\left(i\right)},k-1\right),i=0,1,\cdots ,2n$$

(19) $${\hat{X}}_{k}=\sum_{i=1}^{2n}\varpi_{i}^{\left(m\right)}\chi_{k}^{\left(i\right)}$$

(20) $$P_{XX}=\sum_{i=0}^{2n}\omega_{i}^{\left(c\right)}\left(\chi_{k}^{\left(i\right)}-{\hat{X}}_{k}\right){\left(\chi_{k}^{\left(i\right)}-{\hat{X}}_{k}\right)}^{T}$$

(21) $$P_{k}=P_{XX}+Q_{k}$$

where $P_{XX}$ is the state measurement covariance, $\varpi_{i}^{\left(m\right)}$ and $\varpi_{i}^{\left(c\right)}$ are weight vectors for the mean and covariance, respectively, defined as:

(22) $$\varpi_{0}^{\left(m\right)}=\frac{\lambda }{n+\lambda }$$

(23) $$\varpi_{0}^{\left(c\right)}=\frac{\lambda }{n+\lambda }+\left(1-\alpha^{2}+\beta \right)$$

(24) $$\varpi_{i}^{\left(m\right)}=\varpi_{i}^{\left(c\right)}=\frac{1}{2\left(n+\lambda \right)},i=1,2,\cdots ,2n$$

where the optimal setting of β is β=2, and n is the dimension of the state.

Step 4: Measuring predictions

(25) $$\xi_{k}^{\left(i\right)}=h\left(\chi_{k}^{\left(i\right)},k\right),i=0,1,2,\cdots ,2n$$

(26) $${\hat{Z}}_{k}=\sum_{i=0}^{2n}\varpi_{i}^{\left(m\right)}\xi_{k}^{\left(i\right)}=\sum_{i=0}^{2n}\varpi_{i}^{\left(m\right)}Z_{k}$$

Step 5: Kalman Gain Calculations

(27) $$P_{XZ}=\sum_{i=0}^{2n}\varpi_{i}^{\left(c\right)}\left(\chi_{k}^{\left(i\right)}-{\hat{X}}_{k}\right){\left(\xi_{k}^{\left(i\right)}-{\hat{Z}}_{k}\right)}^{T}$$

(28) $$P_{ZZ}=\sum_{i=0}^{2n}\left(\xi_{k}^{\left(i\right)}-{\hat{Z}}_{k}\right){\left(\xi_{k}^{\left(i\right)}-{\hat{Z}}_{k}\right)}^{T}+R_{k}$$

(29) $$K_{k}=P_{XZ}P_{ZZ}^{-1}$$

where $P_{XZ}$ is the state measurement cross-covariance, $P_{ZZ}$ is the new interest covariance, $K_{k}$ is the Kalman gain.

Step 6: Filter Updates

(30) $${\hat{X}}_{k+1}={\hat{X}}_{k}+K_{k}\left(Z_{k}-{\hat{Z}}_{k}\right)$$

(31) $$P_{k+1}=P_{k}-K_{k}P_{ZZ}K_{k}^{T}$$

Step 7: Repeat Steps 2–6 until all sample points have been calculated.

The standard filtering algorithms described above make Gaussian distribution assumptions about the statistical properties of both process and observation noise. In contrast, the actual noise often does not conform to these assumptions. Such assumptions may lead to bias in the noise estimation and affect the filtering effect. As a result, the Sage–Husa method is used to improve the UKF, leading to the proposal of an adaptive UKF algorithm.

### 2.4. Adaptive UKF Algorithm

**Figure 1:**
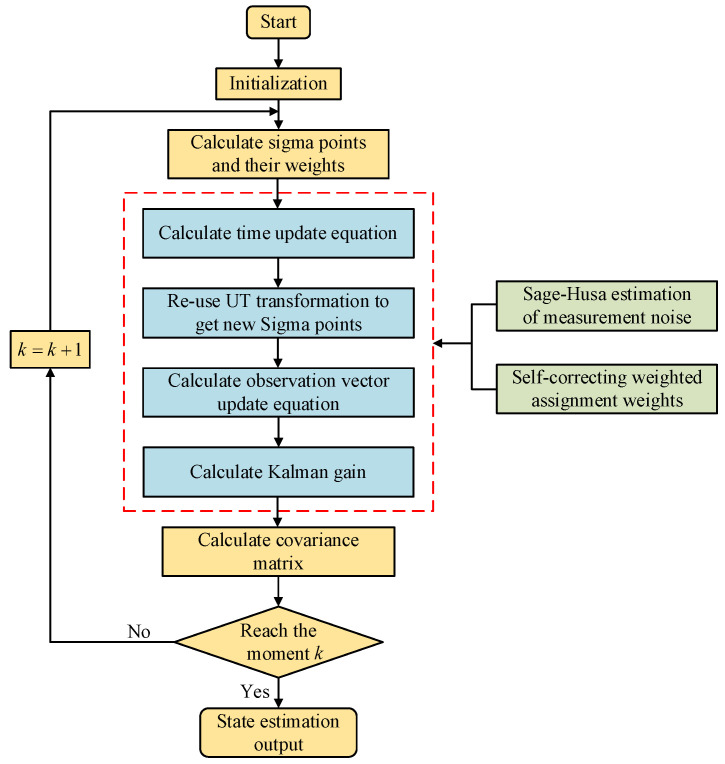
Flowchart of the self-correcting weighted AUKF.

To address the uncertainty of measurement noise in the real process, this work suggests an online update $R_{k}$-based adaptive UKF based on Sage–Husa theory, which reduces the complexity of the algorithm without affecting the accuracy. The flow chart for it is displayed in Figure 1.

In contrast to the conventional UKF algorithm, the AUKF algorithm uses the quantitative noise data to estimate and correct the statistical properties of the measurement noise in real time, using the observation data when recursive filtering, which can reduce the estimation error, inhibit the filtering dispersion and improve the filtering accuracy.

The steps for estimating the measurement noise based on the Sage–Husa algorithm with the system noise time invariant are as follows:

(32) $$\mu_{k}=\frac{1-\vartheta }{1-\vartheta^{k}}$$

(33) $$\varepsilon_{k}=Z_{k}-{\hat{Z}}_{k}-r_{k}$$

(34) $${\hat{r}}_{k}=\left(1-\mu_{k}\right){\hat{r}}_{k-1}+\mu_{k}\left[Z_{k}-\sum_{i=0}^{2n}\omega^{\left(i\right)}Z_{k-1}^{\left(i\right)}\right]$$

(35) $${\hat{R}}_{k}=\left(1-\mu_{k}\right){\hat{R}}_{k-1}+\mu_{k}\left(\varepsilon_{k}\varepsilon_{k}^{T}\right)$$

where $\mu_{k}$ is the proportion of noise that is adjusted in real time according to the system in which it is located and the external situation to strengthen the reliability and trustworthiness of the system, reduce the system noise and external interference, improve the ability to cope with sudden changes in the state, and strengthen the tracking performance. ϑ is the forgetting factor, and its selection range is 0.95<ϑ<0.99.

The weighted fusion algorithm Equation (13) is obtained by substituting Equations (26) and (35) is obtained by substituting Equation (27):

(36) $${\hat{Z}}_{k}^{*}=\sum_{i=0}^{2n}\omega_{i}^{\left(m\right)}\xi_{k}^{\left(i\right)}=\sum_{i=0}^{2n}\omega_{i}^{\left(m\right)}Z_{kw}$$

(37) $$P_{XZ}=\sum_{i=0}^{2n}\omega_{i}^{\left(c\right)}\left(\chi_{k}^{\left(i\right)}-{\hat{X}}_{k}\right){\left(\xi_{k}^{\left(i\right)}-{\hat{Z}}_{k}^{*}\right)}^{T}$$

(38) $$P_{ZZ}=\sum_{i=0}^{2n}\left(\xi_{k}^{\left(i\right)}-{\hat{Z}}_{k}^{*}\right){\left(\xi_{k}^{\left(i\right)}-{\hat{Z}}_{k}^{*}\right)}^{T}+{\hat{R}}_{k}$$

Compared with the standard UKF, the self-correcting weighted AUKF adds the Sage–Husa algorithm and self-correcting weighting to assign the weights of measurement values, adds the Sage–Husa algorithm to update the variance of the measurement noise in real time, and adds the self-correcting weighting to make up for the defects of the traditional weighting algorithm affected by the historical data so as to realize the purpose of the high-precision positioning of the maglev train.

## 3. Experimental Results and Analyses

**Figure 2:**
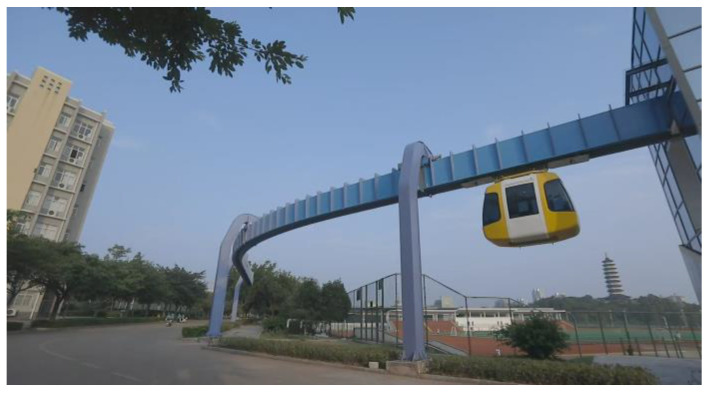
The 60 m maglev train test line.

**Figure 3:**
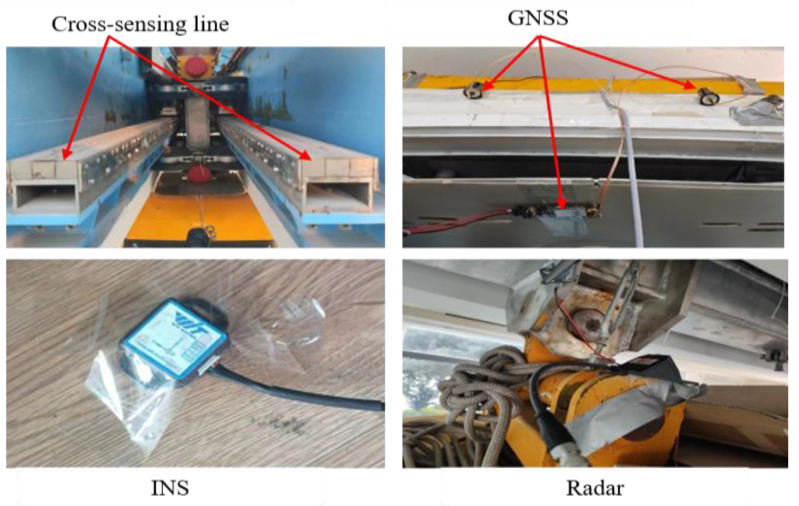
Diagram of the experimental equipment for the 60 m technology validation line.

**Figure 4:**
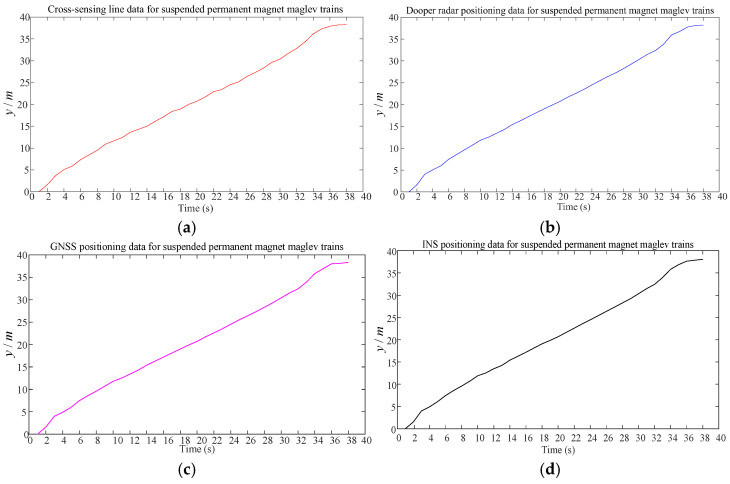
Sensor data acquisition diagram. (**a**) Cross-sensing line data; (**b**) Doppler radar data; (**c**) GNSS data; (**d**) INS data.

The 60 m levitation of the magnet train test line serves as the experimental object. The experiment combines relative and absolute positioning using experimental equipment such as GNSS, Doppler radar, and INS for relative positioning and a cross-sensing line for absolute positioning correction. Figure 2 and Figure 3 display the experimental objects and data-collecting sensors, and Figure 4 displays the data collected by each sensor.

**Figure 5:**
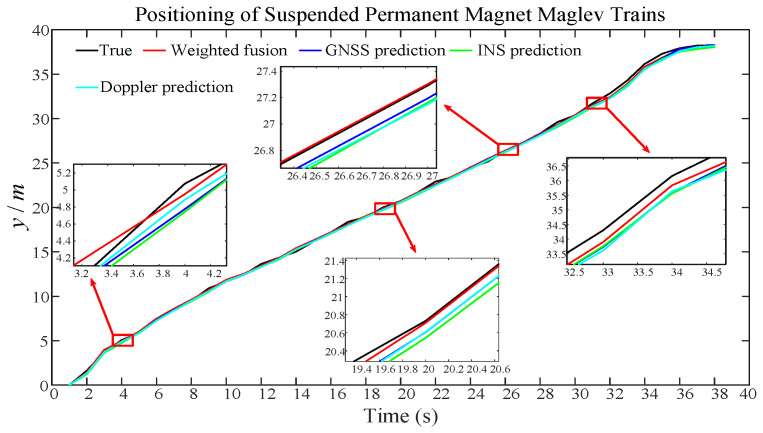
Comparison of self-correcting weighted AUKF and single-sensor AUKF.

To test and validate the self-correcting weighted AUKF algorithm proposed in this paper for the positioning accuracy and error of the maglev train, compared with a single sensor, the prediction results of the self-correcting weighted AUKF algorithm and the single sensor are shown in Figure 5 below.

As can be seen from the comparison plot of the results of self-correcting weighted fusion and single-sensor prediction using AUKF in Figure 5, the prediction results using self-correcting weighted fusion are closer to the real values. The prediction results of single-sensor GNSS, INS, and Doppler radar are slightly farther away in comparison to self-correcting weighted fusion, which suggests that the self-correcting weighted fusion AUKF method proposed in this paper is favorable to the maglev train positioning accuracy improvement.

Error metrics are important in assessing the accuracy and reliability of measurement or estimation results. By analyzing these errors, it is possible to understand the extent to which the estimated values differ from the true values, assess the quality of the measurement or estimation results, and optimize algorithms and models to improve their performance.

(1) Mean Error (ME):

(39) $$ME=\frac{1}{m}\sum_{i=1}^{m}\left|y_{i}-{\hat{x}}_{i}\right|$$

(2) Root Mean Square Error (RMSE):

(40) $$RMSE=\sqrt{\frac{1}{m}\sum_{i=1}^{m}{\left(y_{i}-{\hat{x}}_{i}\right)}^{2}}$$

According to Equations (39) and (40), a comparison of error indicators can be obtained as shown in Table 1 below.

In light of the contrast between the error metrics in Table 1, it can be concluded that the self-corrected weighting reduces the ME by 13.03% and the RMSE by 20.51% compared to a single GNSS throughout the AUKF experiment. The RMSE is reduced by 31.28% concerning the INS, the ME is reduced by 21.94%, and the RMSE is reduced by 27.16% compared to the Doppler radar.

**Figure 6:**
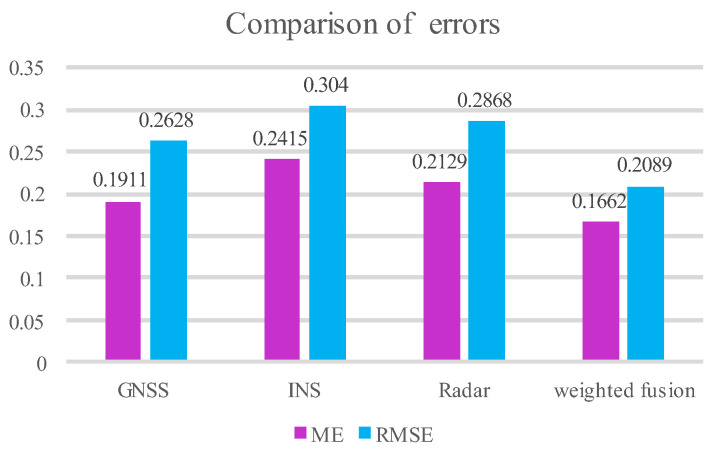
Comparison of self-correcting weighted and single-sensor errors.

**Figure 7:**
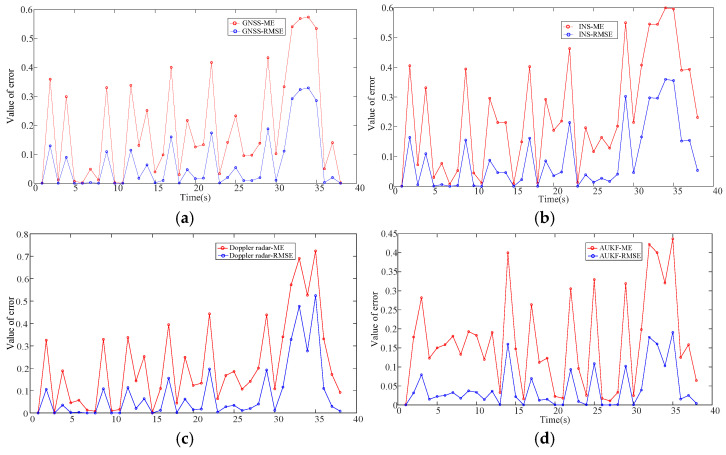
Comparison of the error of each sensor. (**a**) GNSS error plot; (**b**) INS error plot; (**c**) radar error plot; (**d**) weighted fusion error plot.

From the comparison of the error metrics in Table 1, a histogram of the error comparison between the self-corrected weighted fusion and single-sensor prediction results using the AUKF can be obtained, and an error comparison graph is shown in Figure 6 and Figure 7.

As can be seen from the error comparison plots in Figure 6 and Figure 7, the ME and RMSE of the self-corrected weighted fusion are smaller than those of the single-sensor GNSS and INS, as well as the Doppler radar during the experimental prediction process, which indicates there is some degree of reliability in the weighting system that this study recommends.

**Figure 8:**
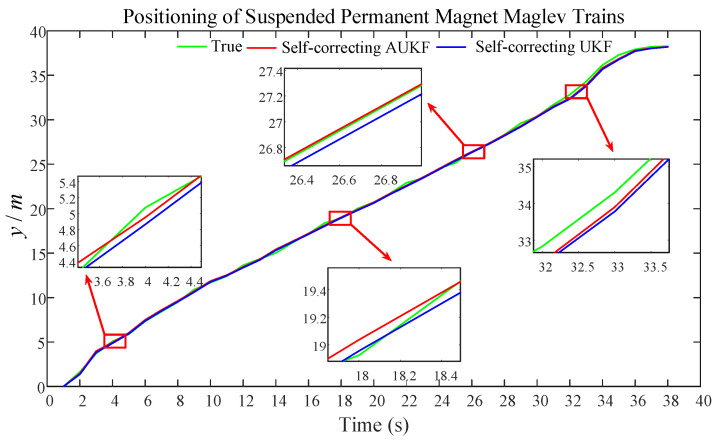
Comparison of self-correcting weighted UKF and self-correcting weighted AUKF.

For verifying the validity and accuracy of the AUKF research method based on self-correcting weighted fusion proposed in this paper, Figure 8 presents a comparison of the prediction outcomes for the self-correcting weighted fusion AUKF and self-correcting weighted fusion UKF.

From Figure 8, the comparison of the prediction results of the self-correcting weighted UKF and the self-correcting weighted AUKF, it can be seen that the curve of the prediction results of the AUKF is closer to the curve of the real value compared with the standard UKF. Then, it can be shown that the predicted positioning results of the AUKF are closer to the real value, which improves the positioning accuracy of the maglev train, thus proving that the mathematical model establishment of the self-correcting weighted fusion AUKF is the correctness, robustness, and accuracy of this algorithm.

To compare the accuracy of the prediction results between the self-correcting weighted UKF and the self-correcting weighted AUKF, a comparison of their error metrics is shown in Table 2.

Based on the comparison of the UKF and AUKF error metrics in Table 2, it can be concluded that the ME of the self-corrected AUKF is reduced by 11.03%, and the RMSE is reduced by 13.32% compared to the self-corrected UKF.

**Figure 9:**
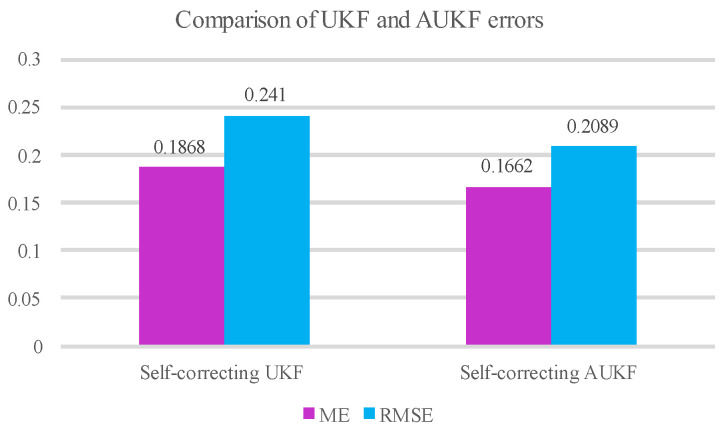
Histogram of UKF and AUKF error comparison.

**Figure 10:**
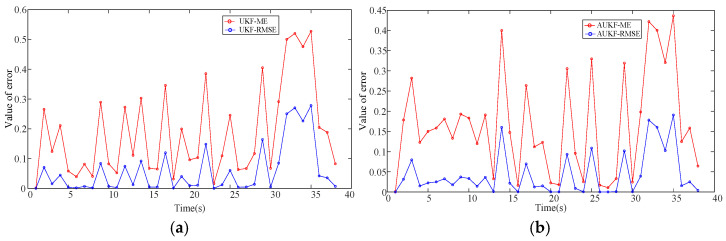
Comparison of UKF and AUKF errors. (**a**) UKF error plot; (**b**) AUKF error plot.

Figure 9 and Figure 10 represent the error comparison histograms and error comparison plots of the self-correcting weighted UKF and self-correcting weighted AUKF, respectively. It is seen that the error curves of the self-correcting weighted AUKF in the two plots are relatively low, which proves that the AUKF is more effective than the UKF and is more suitable for the localization of the maglev train.

## 4. Conclusions

Many references show that the current positioning of maglev trains has problems such as single sensors, noise interference, and fixed traditional weighting weights, which leads to a significant reduction in the positioning accuracy and is not conducive to their safe operation. Therefore, we propose a multi-sensor information fusion positioning method for AUKF maglev trains based on self-correcting weighting. We mainly improve the classical UKF algorithm from two aspects. Firstly, the self-correcting weighting algorithm is used to improve the traditional weighting algorithm, which is susceptible to the influence of historical data, and the weights are fixed. Secondly, the Sage–Husa is used for real-time estimation and correction of measurement noise.

From the comparison graph of experimental prediction results and the error comparison graph, the prediction results of the self-corrected weighted fusion AUKF are closer to the real value, which greatly reduces the positioning error and improves the positioning accuracy. The experimental results show that it is feasible to apply this method to the positioning of maglev trains.

In the method proposed in this paper, the use of the Sage–Husa algorithm only estimates and corrects the measurement noise in real time and ignores the influence of the process noise, but in practice, both the process noise and the measurement noise will have a more serious impact on the positioning accuracy of the maglev train. Therefore, in our future work, we need to further improve the algorithm by considering the effects of both kinds of noise and verifying its effectiveness through experimental simulation.

## Figures and Tables

**Table 1:** Table of error evaluation indexes for single sensor and weighted fusion.

Error Indicators	GNSS	INS	Doppler Radar	Weighted Fusion
ME	0.1911	0.2415	0.2129	0.1662
RMSE	0.2628	0.3040	0.2868	0.2089

**Table 2:** Table of error evaluation indexes for self-correcting UKF and self-correcting AUKF.

Error Indicators	Self-Correcting UKF	Self-Correcting AUKF
ME	0.1868	0.1662
RMSE	0.2410	0.2089

## Data Availability

Data are only available upon request due to restrictions regarding, e.g., privacy and ethics. The data presented in this study are available from the corresponding author upon request. The data are not publicly available due to their relation to other ongoing research.
